# The impact of tumor size on the prognosis and chemotherapy efficacy in stage I/II colon cancer patients

**DOI:** 10.7150/jca.95743

**Published:** 2024-07-22

**Authors:** Jiahe Yang, Shichao Li, Yulu Zhao, Fangyuan Yang, Qian Wang, Lan Ding, Cheng Chen, Xiaoyuan Chu

**Affiliations:** 1 Department of Medical Oncology, Nanjing Jinling Hospital, Affiliated Hospital of Medical School, Nanjing University, Nanjing, China.; 2 Department of Pathology, General Hospital of Xinjiang Military Command, Urumqi, Xinjiang, China.; 3 Department of Medical Oncology, Jinling Hospital, Nanjing Medical University, Nanjing, China.; 4 Department of Medical Oncology, Jinling Hospital, The First School of Clinical Medicine, Southern Medical University, Nanjing, China.; 5 Department of General Surgery, Nanjing Jinling Hospital, Affiliated Hospital of Medical School, Nanjing University, Nanjing, China.

**Keywords:** Colon cancer, Tumor size, Stage I/II, Prognosis, Chemotherapy

## Abstract

**Background:** The impact of tumor size on the survival and chemotherapy reponse of early-stage colon cancer remains unclear. Our study explored the effect of tumor size on overall survival (OS) and postoperative chemotherapy efficacy in patients with stage I/II colon cancer.

**Methods:** Stage I/II colon cancer patients from the Surveillance, Epidemiology and End Results (SEER) database and a China center were extracted as two cohorts respectively. X-tile program was adopted to acquire optimal cutoff points of tumor size (16mm and 49mm). Harrell's concordance index (c-index) and time-dependent receiver operating characteristic curve (ROC) were used to indicate discrimination ability of prognostic factors.

**Results:** Overall, 104,908 and 168 stage I/II postoperative colon cancer patients from SEER database and a China center were eligible, respectively. Kaplan-Meier analysis showed that large tumor size was associated with poor OS in two cohorts. The effect of tumor size on OS gradually decreased as the T stage increased both before PSM (c-index 0.535 for T1N0M0 and 0.506 for T4N0M0, p<0.05) and after PSM (c-index 0.543 for T1N0M0, p<0.05; c-index 0.543 for T4N0M0, p>0.05). Stratified analyses showed that chemotherapy improved the OS rate by 9.5% (chemotherapy vs. non-chemotherapy: 83.5% vs. 73.0%) or 12.8% (chemotherapy vs. non-chemotherapy: 85.7% vs. 72.9%) before and after PSM in T2N0M0 patients with tumor size >49 mm, but not in T1N0M0. The survival benefit provided by chemotherapy for T2N0M0 patients with large tumor was also validated in the Chinese cohort.

**Conclusions:** Large tumor size was a risk factor for stage I/II colon cancer, especially for T1N0M0. Tumor size could serve as a complementary factor guiding postoperative chemotherapy for T2N0M0 patients.

## Background

Colon cancer is one of the most common malignant tumors in clinic, with high morbidity and mortality 1. In recent years, with the increase of economy level and health awareness, and the popularization of health examinations, about 75% of colon cancers were diagnosed at early stage 2. Although radical surgery plays a critical role in the management of these patients, up to one quarter of node-negative colon cancers (i.e. stage I/II) will recur after surgery 3. Thus, finding clinical features to identify stage I/II patients with distinct prognoses is imperative.

Tumor size is defined as the largest horizontal tumor diameter 4. American Joint Committee on Cancer (the eighth edition) adds tumor size to the staging manual of many tumors, but not in colon cancer. For colon cancer, T stage is defined by the depth of tumor invasion rather than tumor size in the staging system. This may be attributed to the unclear relationship between tumor size and colon cancer. Previous studies have shown that smaller tumor size was correlated with poor prognosis in T4b colon cancer 5, 6, suggesting a possible link between small tumor size and more biologically aggressive phenotype 6. Another explanation was that clinicians tended to treat large tumors more aggressively, resulting in better survival of patients with large tumors 7. On the contrary, some studies have demonstrated that larger tumor size predicted shorter survival time for T1 colon cancer 8. Most of the studies incorporated patients with lymph node metastasis, but for node-negative colon cancer (stage I/II), the role of tumor size on prognosis needs further exploration.

According to the National Comprehensive Cancer Network (NCCN) guidelines (version 2.2023) 9, stage I (T1-2, N0, M0) colon cancer patients are suggested with observation after transabdominal resection, and high-risk stage II (T3-4, N0, M0) patients are recommended to receive chemotherapy following surgery. In addition to T stage, identifying other critical prognostic factor may help guiding postoperative chemotherapy plan for stage I/II colon cancer 10.

In this study, we used the Surveillance, Epidemiology, and End Results (SEER) database and an external validation cohort to analyze the impact of tumor size on the prognosis of stage I/II postoperative patients and further explored whether tumor size could be associated with chemotherapy efficacy for certain subgroups of early-stage colon cancer patients.

## Methods

### Data source and exclusion criteria

Data used in the present study were extracted from the National Cancer Institute's Surveillance, Epidemiology, and End Results (SEER) program database, which contained information from population-based cancer registries on patient demographics, cancer incidence, treatment, and outcomes (https://seer.cancer.gov). The database we selected was SEER Research Plus Data, 17 registries, Nov 2021 Sub (2000-2019).

**Figure 1:**
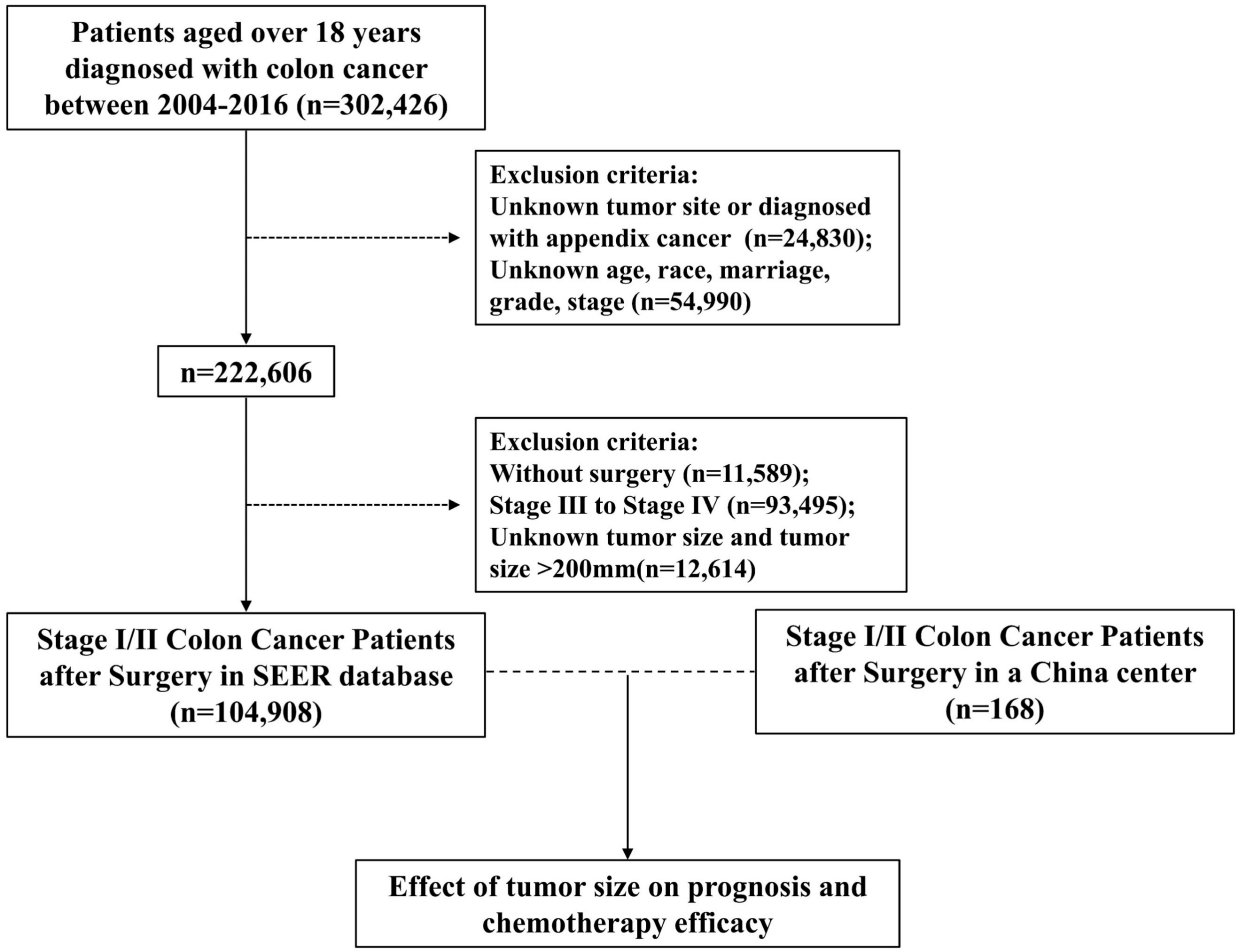
Flow diagram of eligible patients from the SEER Database and a China center.

Data of patients ≥18 years old and diagnosed with colon cancer between 2004 and 2016 were obtained from SEER database. Patients with reports presented in the form of death certificates or autopsy only were not enrolled, as were those without pathologically confirmed diagnoses. Figure 1 depicts the data selection process. To definite the location of tumors, tumor sites such as appendix, overlapping lesions of colon or colon not otherwise specified (NOS) were excluded. To enhance the validity and authenticity of the present study, patients with missing values on crucial covariates such as age, race, marriage, grade and stage were excluded. Then we chose stage I/II patients who underwent surgery, excluding patients with unknown tumor size or tumor size over 200mm. Finally, 104,908 patients were included in our study.

Data used in the validation cohort were extracted from General Hospital of Xinjiang Military Command between 2013 and 2020. The inclusion criteria were the same as SEER database above. After exclusion, a total of 168 patients with stage I/II colon cancer were identified (Figure 1). Descriptive analysis of the validation cohort was provided in Table S1.

### Statistical analysis

All analyses were performed using the R statistical software (version 4.3.0) by Bell Laboratories in New Jersey, United States. Demographic and clinical features were analyzed with a chi-square (χ2) test. Survival curves were generated using Kaplan-Meier estimates. The X-tile program was used to generate the optimal tumor size cutoff points with minimum P values from chi-square tests 11. In order to adjust for differences in baseline characteristics and to minimize bias, propensity score matching with a ratio of 1:1:1 was performed on the MENGTE platform (https://mengte.pro/psm). Both univariate and multivariate Cox proportional hazard models were used to identify variables associated with survival, and the results are presented as hazard ratios (HRs) and 95% confidence intervals (95% CIs). The discriminative ability of tumor size was evaluated by using the Harrell's concordance index (c-index) and time-dependent receiver operating characteristic curve (ROC) 12, 13. A two-sided P < 0.05 was considered statistically significant.

## Results

### Patient characteristics

**Figure 2:**
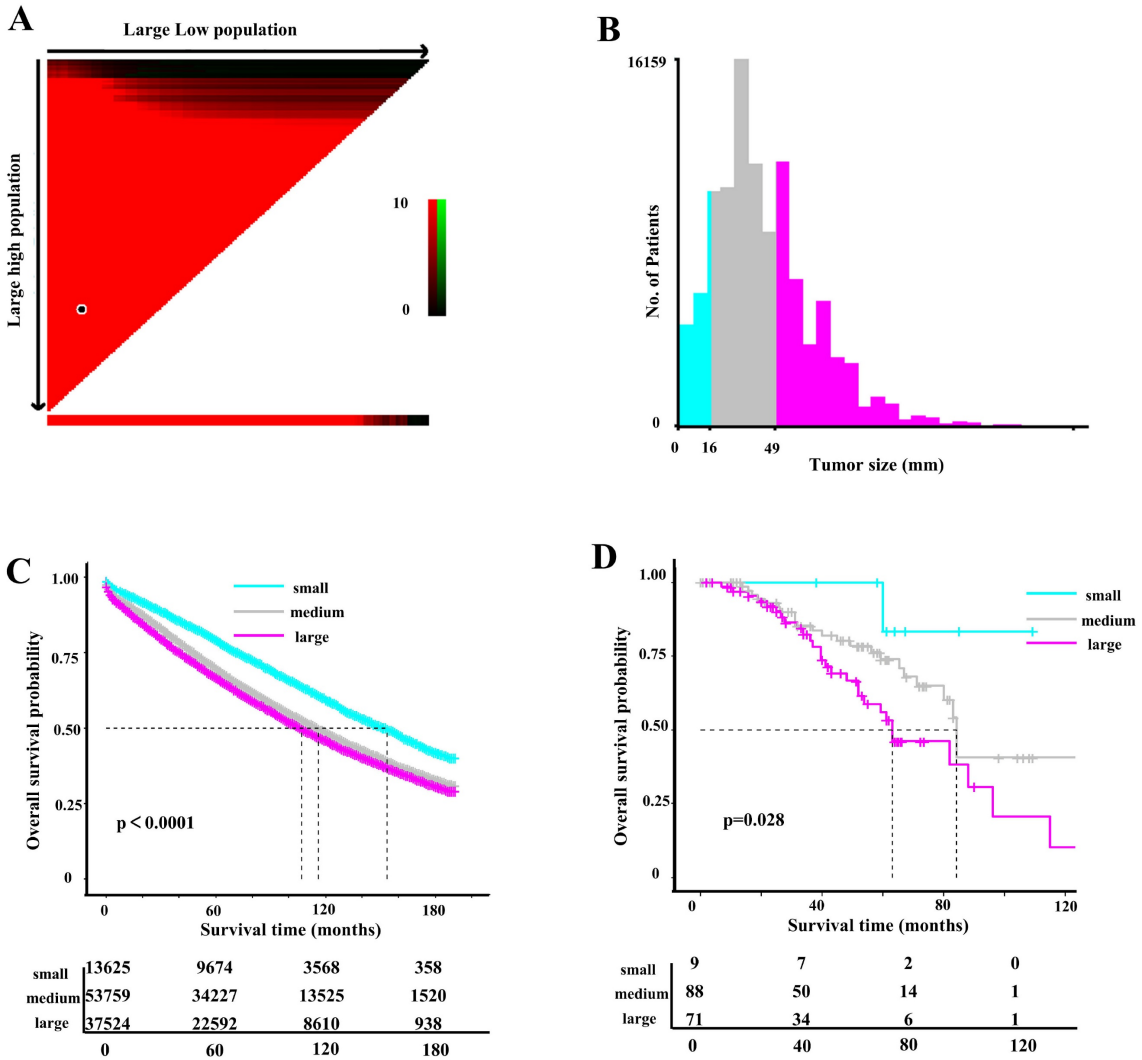
(A) The X-tile plot, (B) histogram and (C) OS curve in X-tile analysis of survival data from SEER database. (D) Kaplan-Meier analysis of OS between three tumor size groups in the external validation cohort.

A total of 104,908 eligible cases from SEER database with stage I/II colon cancer were finally included in this analysis (Figure 1). Based on a cutoff of 16mm and 49mm determined by X-tile analysis (Figure 2A and 2B), we divided 104,908 patients into three subsets, of which 13625 (13.0%) patients were assigned as the small tumor group (tumor size ≤16mm), 53759 patients (51.2%) were classified as medium tumor group (16mm<tumor size ≤49mm) and 37524 patients (35.8%) were classified as large tumor group (tumor size >49 mm). The clinicopathological characteristics of patients based on tumor size were presented in Table 1. Patients with tumors larger than 16 mm tended to be elder, right-sited and had higher T stage and grade. The ratio of patients receiving postoperative chemotherapy was higher in medium and large tumor groups.

Since there were substantial differences in characteristics between different tumor size groups, propensity score matching (PSM) was performed to reduce the differences (Table 1). All baseline characteristics were balanced after PSM, except for primary site.

### Tumor size was related to prognosis in stage I/II patients

Univariate and multivariate Cox regression models revealed that larger tumor size was associated with higher risk of overall death (Table 2). Kaplan-Meier analyses showed that the small tumor group had the best OS, while the large tumor group had the worst survival (P < 0.001) before and after PSM (Figure 2C and Figure S1). To further validate the cutoff value, 168 patients from a Chinese center (General Hospital of Xinjiang Military Command) were included as an external validation cohort. The medium tumor group were associated with worse OS compared with small tumor group, and large tumor group had an even worse OS in the validation cohort (P=0.028) (Figure 2D).

### Prognostic value of tumor size varied in different T stages

To further explore the interaction between tumor size and other risk factors, we performed a subgroup analysis. Based on this analysis, larger tumor size was a risk factor for the majority of subgroup cases, except for SRCC, grade IV and T4 patients (Table S2). Patients over 65 years old with tumor size larger than 49mm had the shortest median survival time, while the survival probability of patients ≤65 years old was larger than 0.5 in any tumor size group.

Notably, the impact of tumor size on death risk decreased with the increase of T-stage. Tumor size was associated with the greatest impact on death risk in T1N0M0 colon cancer (medium refer to small: HR=1.254, p<0.001; large refer to small: HR=1.316, p<0.001), but was no longer a prognosis predictor in T4N0M0 colon cancer (medium refer to small: HR=1.1, p>0.05; large refer to small: HR=1.02, p>0.05) (Table S2).

Furthermore, c-index and time-dependent ROC were used to evaluate the predictive ability of clinical factors before and after PSM (Table 3 and Table S3). Among all stage I/II colon cancer patients, T stage was the best predictor for OS (C-index 0.558 before PSM, C-index 0.533 after PSM), followed by tumor size (C-index 0.534 before PSM, C-index 0.519 after PSM). Subgroup analyses based on T stage showed that in patients with T1N0M0 colon cancer, tumor size outperformed any other factors in predicting OS both before and after PSM. However, once T stage increased, the predictive ability of tumor size gradually reduced (Table 3 and Table S3). Taken together, these results indicated that large tumor size was a risk factor for stage I/II colon cancer patients, especially for T1N0M0 patients.

### The influence of tumor size on chemotherapy efficacy in stratified T stages

**Figure 3:**
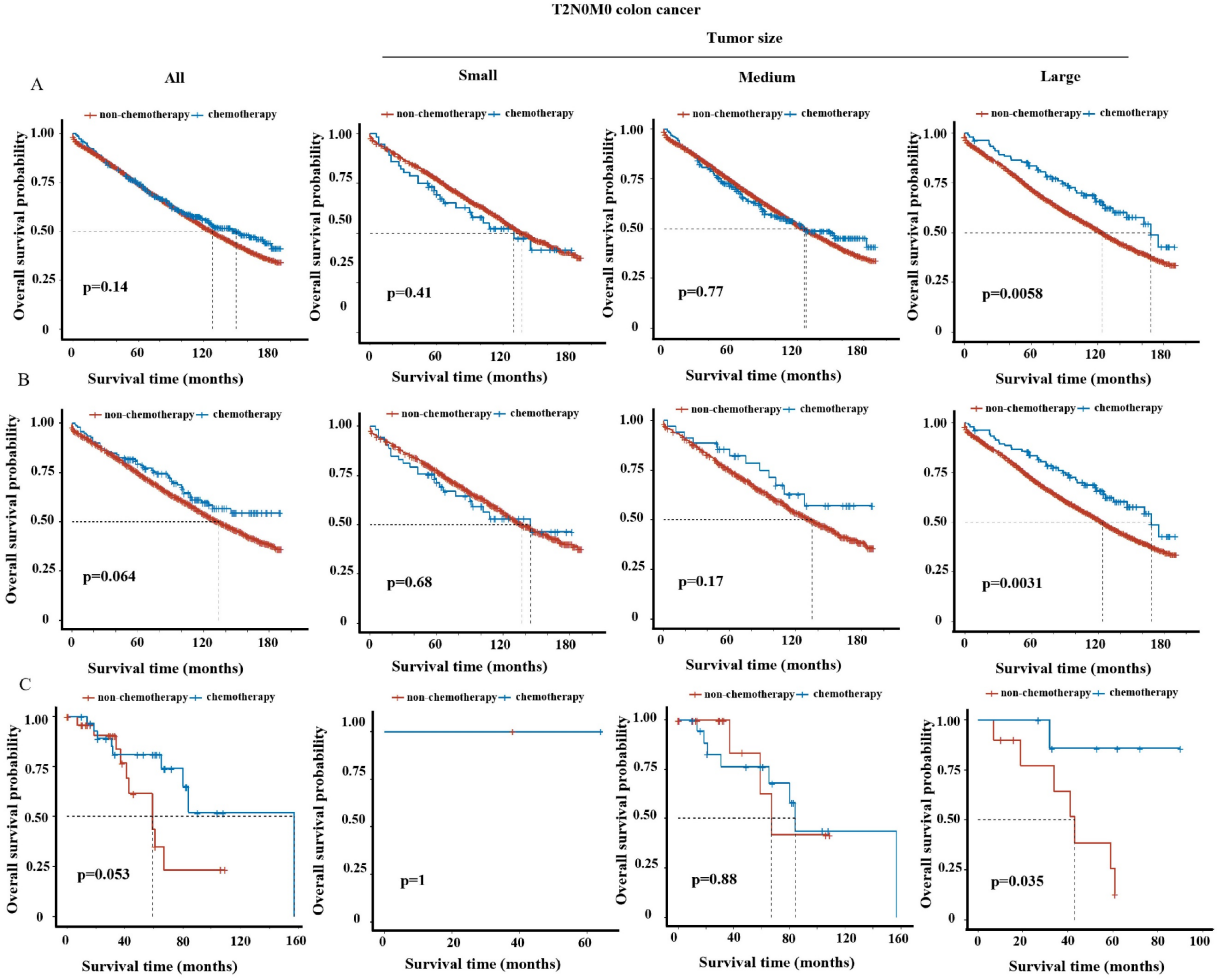
OS curve of chemotherapy group vs. non-chemotherapy group in T2N0M0 patients among overall and three tumor size groups in the SEER cohort (A) before and (B) after PSM and (C) in the external validation cohort.

We tended to further explore whether tumor size could be a supplementary factor guiding postoperative treatment. Kaplan-Meier analysis revealed significant improvement of survival in overall T3N0M0 or T4N0M0 patients (i.e. stage II) upon chemotherapy treatment (Figure S2B-C and S3B-C), but not in overall T1N0M0 (Figure S2A and S3A) or T2N0M0 patients (Figure 3A and 3B) (i.e. stage I) both before and after PSM, consistent with NCCN guidelines 9. We then analyzed the impact of tumor size on the efficacy of chemotherapy in stratified T stage. For T3N0M0 and T4N0M0 patients, the benefits of chemotherapy were observed in all three tumor size groups (Figure S2B-C and S3B-C). For T1N0M0 patients, chemotherapy did not improve but even decrease the survival in small and medium tumor group (Figure S2A and S3A), which may due to the toxic side-effect of chemotherapy. Intriguingly, for T2N0M0 patients, postoperative chemotherapy significantly improved survival (p<0.05) in large tumor group, but not in small or medium tumor group in the SEER cohort before and after PSM (Figure 3A-B). The improvement of 5-year OS rate resulted from chemotherapy was 9.5% (chemotherapy vs. non-chemotherapy: 83.5% vs. 73.0%) before PSM or 12.8% (chemotherapy vs. non-chemotherapy: 85.7% vs. 72.9%) after PSM for T2N0M0 patients with large tumors. In line, chemotherapy also provided survival benefit for T2N0M0 patients with large tumors in the validation cohort (Figure 3C).

## Discussion

In this large population-based study, we investigated the effect of tumor size on postoperative survival in patients with early-stage colon cancer. We found that larger tumor size was associated with poorer prognosis in stage I/II patients. This conclusion is consistent with some of the previous studies 14. In contrast, Huang et al. found a negative association between tumor size and prognosis in stage II colon cancer patients 15. The reason may be that they chose only Caucasian race and focused on the cancer specific survival which was different from long-term overall survival in our study.

T stage has a unique definition in colon cancer, which reflects the depth of local invasion rather than tumor size. Studies have shown that the prognosis of colon cancer patients at different T stages was affected by tumor size differently. In patients with T1 colon cancer, one study showed that the larger the tumor size, the worse the patient's prognosis in partial patients 8. On the other hand, multiple studies demonstrated that smaller tumor size led to worse prognosis in T4 colon cancer 6, 16. In our study, stratified analysis also showed that the hazard ratio was highest in T1N0M0 patients and subsequently decreased as T stage progressed. In T4N0M0 patients, tumor size was no longer an independent factor affecting prognosis. As the depth of tumor infiltration deepens, the ability of tumor size to influence prognosis weakens. Some researchers have suggested that small tumors that reach the serosa and beyond may reflect vertical growth patterns and early-acquired metastatic potential 17, 18. Our study focused on patients without any lymph node metastasis or distant metastasis, thus tumors with strong invasive metastatic capacity were excluded, and the findings above were not observed in our T4N0M0 patients. In addition, different pathological features may also affect the capacity of tumor size to predicate prognosis. Our result showed that the predictive ability of tumor size weakened in mucinous adenocarcinoma and signet-ring cell adenocarcinoma. In mucinous adenocarcinoma, the secreted extracellular mucus may enhance the aggressiveness of tumor cells, and lessen the ability of tumor size to predict prognosis 19.

In our study, Kaplan-Meier analysis showed that overall T1N0M0 patients with colon cancer had no benefit from postoperative chemotherapy, and chemotherapy even resulted in shorter survival in T1N0M0 patients with small tumor size. We speculated that T1N0M0 patients were less likely to have residual tumor cells or circulating tumor cells after surgery, therefore would benefit less from chemotherapy. Instead, side effects associated with chemotherapy may even lead to shorter OS in this group of patients. Thus, our results supported the NCCN guidelines of suggesting only postoperative observation for T1N0M0 patients.

However, according to NCCN guidelines, T2N0M0 colon cancer patients are suggested with only observation after surgery as same as T1N0M0 patients. Intriguingly, our study showed that chemotherapy could benefit the prognosis of the T2N0M0 patients with large tumor size. To our knowledge, this is the first time to establish a link between tumor size and chemotherapy efficacy in early-stage colon cancer. Our findings suggested the use of postoperative chemotherapy for T2N0M0 patients with large tumor size in the clinical practice.

Our study also has a few limitations. First, this study was a retrospective study and still suffered from miscoding and selection bias. Since SEER database collected data from different registries across the United States, consistency in the processing and interpretation of pathological specimens was not guaranteed, leading to bias due to inter-measurer variability. Second, we will need further validation in prospective clinical trials.

## Conclusions

Our study clarifies the interrelationship between tumor size and T stage in the evaluation of OS for stage I/II colon cancer patients. Moreover, we propose tumor size as a complementary factor guiding postoperative chemotherapy for T2N0M0 patients, better improving long-term survival of these patients.

## Supplementary Material

Supplementary figures and tables.

## Figures and Tables

**Table 1:** The clinicopathological characteristics of stage I/II patients based on tumor size

Character	The pre-PSM cohort				The post-PSM cohort			
	Tumor size			p-value	Tumor size			p-value
	≤16mm	16mm-49mm	>49mm		≤16mm	16mm-49mm	>49mm	
	n=13625	n=53759	n=37524		n=4809	n=4809	n=4809	
Gender								
Male	7113(52.2)	26023(48.4)	18351(48.9)	<0.001	2465 (51.258)	2470 (51.362)	2503 (52.048)	0.701
Female	6512(47.8)	27736(51.6)	19173(51.1)		2344 (48.742)	2339 (48.638)	2306 (47.952)	
Race								
White	11063(81.2)	44271(82.4)	30943(82.5)	<0.001	3951 (82.158)	3903 (81.160)	3949 (82.117)	0.31
Black	1420(10.4)	5281(9.8)	3903(10.4)		444 (9.233)	479 (9.960)	496 (10.314)	
Others	1142(8.4)	4207(7.8)	2678(7.1)		414 (8.609)	427 (8.879)	364 (7.569)	
Age								
≤65	5429(39.8)	16782(31.2)	12993(34.6)	<0.001	1656 (34.435)	1659 (34.498)	1689 (35.122)	0.737
>65	8196(60.2)	36977(68.8)	24531(65.4)		3153 (65.565)	3150 (65.502)	3120 (64.878)	
Histology								
Classical adenocarcinoma	12997(95.4)	49335(91.8)	31425(83.7)	<0.001	4449 (92.514)	4434 (92.202)	4465 (92.847)	0.483
Mucinous adenocarcinoma	566(4.2)	4031(7.5)	5426(14.5)		331 (6.883)	339 (7.049)	313 (6.509)	
Signet-ring cell carcinoma	32(0.2)	200(0.4)	283(0.8)		15 (0.312)	22 (0.457)	12 (0.250)	
Other types	30(0.2)	193(0.4)	390(1)		14 (0.291)	14 (0.291)	19 (0.395)	
Grade								
I (Well)	3033(22.3)	5425(10.1)	3026(8.1)	<0.001	769 (15.991)	792 (16.469)	820 (17.051)	0.157
II (Moderately)	9544(70)	41910(78)	26649(71)		3554 (73.903)	3480 (72.364)	3520 (73.196)	
III (Poorly)	931(6.8)	5648(10.5)	6798(18.1)		434 (9.025)	477 (9.919)	416 (8.650)	
IV (Undifferentiated)	117(0.9)	776(1.4)	1051(2.8)		52 (1.081)	60 (1.248)	53 (1.102)	
T stage								
T1	9641(70.8)	6633(12.3)	835(2.2)	<0.001	832 (17.301)	832 (17.301)	832 (17.301)	1
T2	2527(18.5)	16306(30.3)	4510(12)		2524 (52.485)	2523 (52.464)	2524 (52.485)	
T3	1314(9.6)	27872(51.8)	26373(70.3)		1314 (27.324)	1315 (27.345)	1315 (27.345)	
T4	143(1)	2948(5.5)	5806(15.5)		139 (2.890)	139 (2.890)	138 (2.870)	
Primary site								
Right colon	7637(56.1)	34024(63.3)	25974(69.2)	<0.001	2902 (60.345)	2912 (60.553)	3024 (62.882)	0.018
Left colon	5988(43.9)	19735(36.7)	11550(30.8)		1907 (39.655)	1897 (39.447)	1785 (37.118)	
Chemotherapy								
No/Unknown	13272(97.4)	49188(91.5)	31571(84.1)	<0.001	4546 (94.531)	4511 (93.803)	4531 (94.219)	0.31
Yes	353(2.6)	4571(8.5)	5953(15.9)		263 (5.469)	298 (6.197)	278 (5.781)	

**Table 2:** Univariable and multivariable analysis of overall survival for stage I/II patients

Variables	The pre-PSM cohort							The post-PSM cohort					
	Univariate analysis				Multivariate analysis			Univariate analysis			Multivariate analysis		
	HR		95% CI	p-value	HR	95% CI	p-value	HR	95% CI	p-value	HR	95% CI	p-value
Gender													
Male	Ref				Ref			Ref			Ref		
Female	0.9643		0.948-0.981	<0.001	0.869	0.854-0.884	<0.001	0.896	0.854-0.940	<0.001	0.838	0.799-0.880	<0.001
Race													
White	Ref				Ref			Ref			Ref		
Black	0.8848		0.859-0.912	<0.001	1.087	1.055-1.120	<0.001	0.994	0.917-1.077	0.88	1.165	1.075-1.263	<0.001
Others	0.6784		0.654-0.704	<0.001	0.739	0.712-0.767	<0.001	0.664	0.601-0.734	<0.001	0.744	0.6720.822	<0.001
Age													
≤65	Ref				Ref			Ref			Ref		
>65	3.72		3.632-3.81	<0.001	3.595	3.508-3.684	<0.001	3.899	3.649-4.165	<0.001	3.883	3.631-4.152	<0.001
Histology													
Classical adenocarcinoma	Ref				Ref			Ref			Ref		
Mucinous adenocarcinoma	1.17		1.138-1.203	<0.001	1.051	1.021-1.081	<0.001	1.227	1.123-1.342	<0.001	1.105	1.011-1.208	0.028
Signet-ring cell carcinoma	1.233		1.097-1.386	<0.001	1.034	0.919-1.164	0.577	1.049	0.676-1.627	0.831	1.016	0.653-1.581	0.943
Other types	1.377		1.236-1.534	<0.001	1.128	1.010-1.260	0.032	1.526	1.059-2.197	0.023	1.275	0.879-1.848	0.2
Grade													
I (Well)	Ref				Ref			Ref			Ref		
II (Moderately)	1.112		1.080-1.145	<0.001	1.026	0.996-1.056	0.089	0.998	0.935-1.066	0.963	1.003	0.939-1.071	0.937
III (Poorly)	1.333		1.287-1.381	<0.001	1.114	1.074-1.156	<0.001	1.254	1.141-1.377	<0.001	1.130	1.020-1.242	0.012
IV (Undifferentiated)	1.377		1.286-1.475	<0.001	1.115	1.039-1.196	0.002	1.328	1.067-1.652	0.0109	1.26	1.010-.5749	0.041
Tumor size													
≤16mm	Ref				Ref			Ref			Ref		
16-49mm	1.409		1.367-1.451	<0.001	1.155	1.115-1.195	<0.001	1.113	1.049-1.180	<0.001	1.134	1.069-1.203	<0.001
>49mm	1.544		1.497-1.592	<0.001	1.205	1.160-1.251	<0.001	1.143	1.077-1.213	<0.001	1.179	1.111-1.251	<0.001
T stage													
T1	Ref				Ref			Ref			Ref		
T2	1.229		1.192-1.267	<0.001	1.062	1.027-1.099	<0.001	1.068	0.997-1.144	0.063	1.024	0.955-1.097	0.514
T3	1.464		1.425-1.504	<0.001	1.304	1.262-1.348	<0.001	1.274	1.183-1.372	<0.001	1.25	1.1609-1.350	<0.001
T4	2.162		2.086-2.242	<0.001	2.152	2.063-2.245	<0.001	1.793	1.561-2.059	<0.001	1.96	1.704-2.259	<0.001
Primary site													
Right colon	Ref				Ref			Ref			Ref		
Left colon	0.8415		0.826-0.857	<0.001	1.029	1.009-1.048	0.003	0.854	0.812-0.897	<0.001	1.004	0.955-1.056	0.866
Chemotherapy													
No/Unknown	Ref				Ref			Ref			Ref		
Yes	0.599		0.580-0.620	<0.001	0.662	0.640-0.686	<0.001	0.697	0.623-0.780	<0.001	0.769	0.684-0.864	<0.001
													

**Table 3:** Discriminatory ability of clinicopathological factors in predicting survival in stage I/II colon cancer

Variables	ALL		T1N0M0		T2N0M0		T3N0M0		T4N0M0	
	C-index	AUC	C-index	AUC	C-index	AUC	C-index	AUC	C-index	AUC
Histology	0.509	0.512	0.505	0.507	0.507	0.509	0.506	0.506	0.508	0.503
Grade	0.520	0.524	0.509	0.512	0.507	0.509	0.510	0.510	0.524	0.526
Tumor size	0.534	0.542	0.535	0.543	0.510	0.515	0.502	0.500	0.506	0.487
T stage	0.558	0.576	—	—	—	—	—	—	—	—
Primary site	0.517	0.482	0.527	0.472	0.527	0.469	0.512	0.488	0.506	0.494

## Abbreviations

SEER: The surveillance, epidemiology, and end results
NCCN: National comprehensive cancer network
OS: Overall survival
HR: Hazard ratios
AUC: The area under the receiver operator characteristic curves
C-index: The concordance index
ROC: Receiver operating characteristic
CI: Confidence interval
