# Supplementary material for: The impact of tumor size on the prognosis and chemotherapy efficacy in stage I/II colon cancer patients
Source: J Cancer. 2024 Jul 22;15(15):5020–7. doi: 10.7150/jca.95743 (PMC11310877; doi:10.7150/jca.95743)
Supplement: Supplementary file 1 — Supplementary figures and tables. [file jcav15p5020s1.pdf]

Supplementary materials

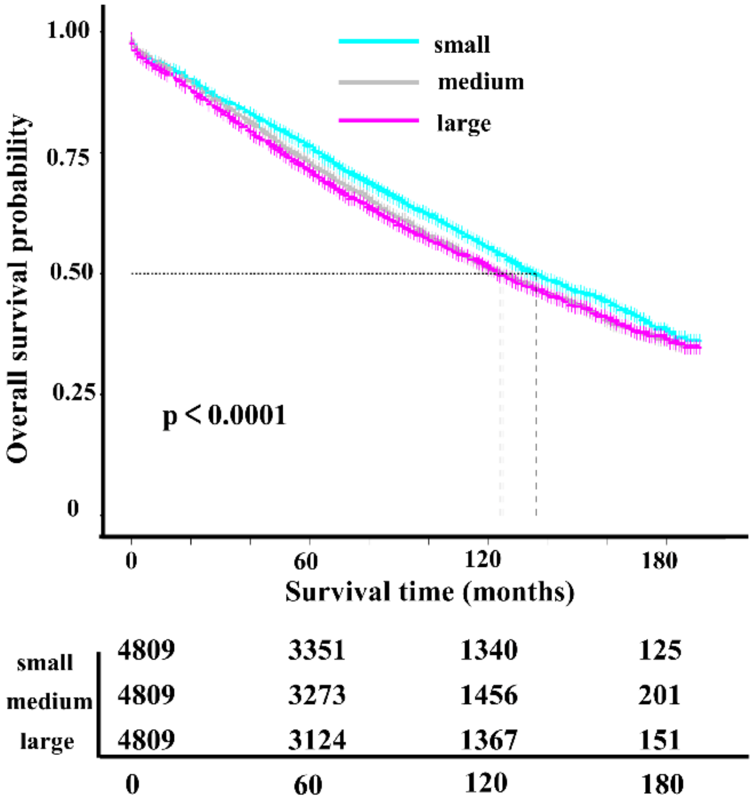

Figure S1. Kaplan-Meier analysis of OS between three tumor size groups from SEER database after PSM.

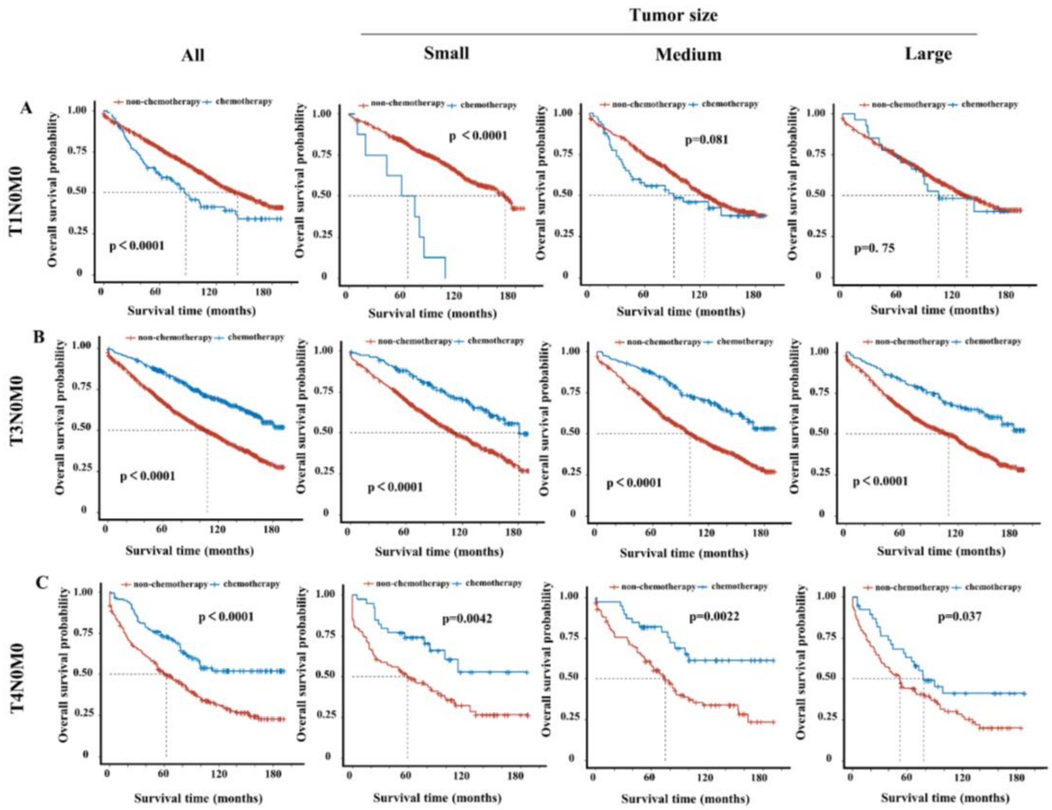

Figure S2. OS curve of chemotherapy group vs. non-chemotherapy group before PSM in (A) T1N0M0 patients, (B) T3N0M0 patients and (C) T4N0M0 patients among overall and three tumor size groups.

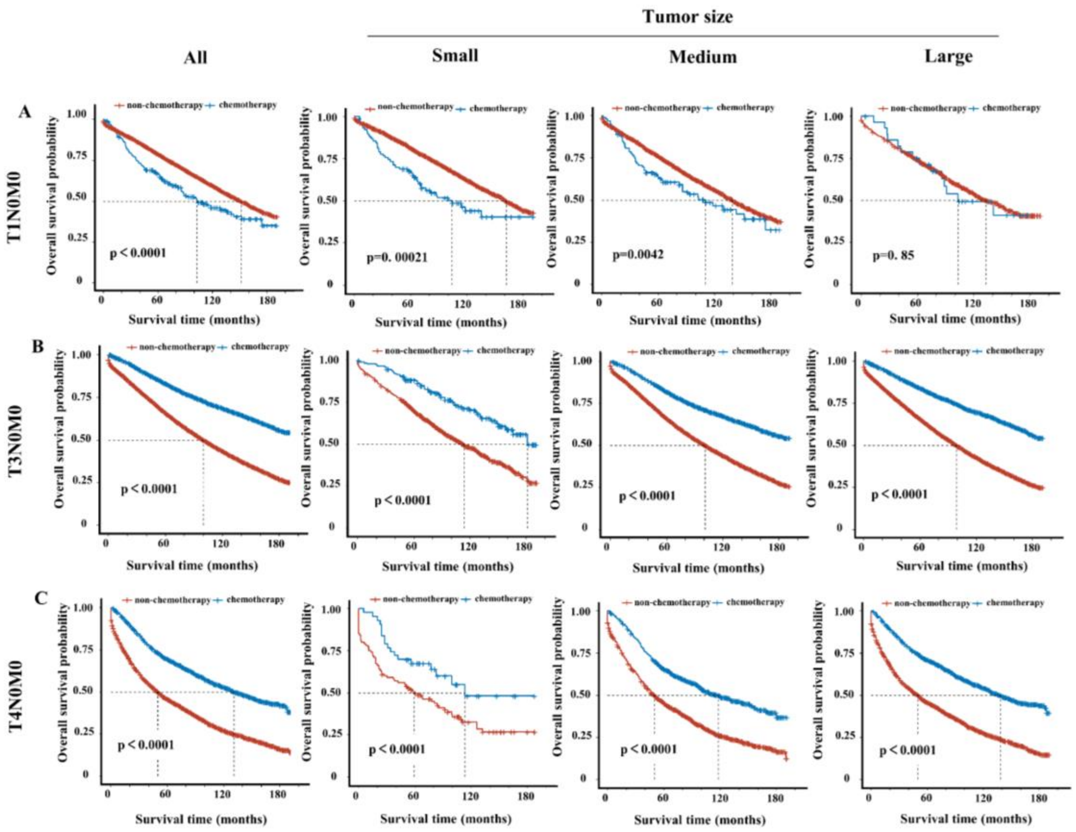

Figure S3. OS curve of chemotherapy group vs. non-chemotherapy group after PSM in (A) T1N0M0 patients, (B) T3N0M0 patients and (C) T4N0M0 patients among overall and three tumor size groups.

Table S1. Descriptive analysis of the validation cohort

Variables	cases(n=168)	percentage
Gender		
Male	83	49.41%
Female	85	50.60%
Age		0.00%
≤65	79	47.02%
> 65	89	52.98%
Histology		0.00%
Classical adenocarcinoma	146	86.91%
Other types	22	13.10%
Grade		0.00%
I/II	136	80.95%
III/IV	32	19.05%
Tumor size		0.00%
≤16mm	9	5.36%
16-49mm	88	52.38%
>49mm	71	42.26%
T stage		0.00%
T1	10	5.95%
T2	55	32.74%
T3	83	49.41%
T4	20	11.91%
Chemotherapy		0.00%
No/Unknown	62	36.91%
Yes	106	63.10%

Table S2. Subgroup analyses of OS between three tumor size groups

Character	Median OS (months)			Hazard ratio (95%CI)	p-value	Hazard ratio (95%CI)	p-value
	small tumor	medium tumor	large tumor	medium refer to small		large refer to small	
Gender							
Male	148	111	109	1.42 (1.363-1.480)	<0.001	1.462 (1.401-1.526)	<0.001
Female	159	121	106	1.407 (1.347-1.469)	<0.001	1.636 (1.565-1.711)	<0.001
Race							
White	149	112	103	1.414 (1.369-1.461)	<0.001	1.553 (1.501-1.606)	<0.001
Black	154	129	122	1.286 (1.170-1.414)	<0.001	1.374 (1.246-1.514)	<0.001
Others	158	154	151	1.524 (1.346-1.726)	<0.001	1.666 (1.464-1.896)	<0.001
Age							
≤65	NA	NA	NA	1.346 (1.252-1.447)	<0.001	1.563 (1.452-1.682)	<0.001
＞65	110	87	76	1.313 (1.271-1.357)	<0.001	1.509 (1.459-1.561)	<0.001
Histology							
Classical adenocarcinoma	157	118	109	1.412 (1.369-1.457)	<0.001	1.543 (1.494-1.593)	<0.001
Mucinous adenocarcinoma	124	101	100	1.217 (1.074-1.379)	0.00209	1.231 (1.088-1.392)	<0.001
Signet-ring cell carcinoma	135	107	100	1.177 (0.6954-1.991)	0.544	1.269 (0.7589-2.122)	0.364
Other types	139	110	73	1.393 (0.7448-2.604)	0.2997	2.085 (1.1384-3.819)	0.0173
Grade							
I (Well)	157	125	117	1.375 (1.282-1.475)	<0.001	1.488 (1.378-1.608)	<0.001
II (Moderately)	156	117	110	1.408 (1.359-1.459)	<0.001	1.506 (1.451-1.563)	<0.001
III (Poorly)	132	104	95	1.312 (1.184-1.455)	<0.001	1.458 (1.317-1.615)	<0.001
IV (Undifferentiated)	107	104	94	1.058 (0.7933-1.411)	0.7011	1.302 (0.9821-1.726)	0.0666
T stage							

T1	161	139	133	1.254(1.193-1.318)	<0.001	1.316(1.181-1.465)	<0.001
T2	137	126	126	1.127(1.057-1.202)	<0.001	1.161(1.079-1.250)	<0.001
T3	125	110	110	1.113(1.027-1.205)	0.00872	1.113(1.028-1.206)	0.00839
T4	81	75	64	1.1 (0.8837-1.368)	0.394	1.02 (0.8216-1.267)	0.857
Primary site							
Right colon	137	110	105	1.334 (1.284-1.386)	<0.001	1.415 (1.361-1.471)	<0.001
Left colon	172	131	115	1.48 (1.410-1.554)	<0.001	1.692 (1.608-1.781)	<0.001

Abbreviations: OS, overall survival; CI, confidence interval; NA, not available.

Table S3. Subgroup analyses of OS between three tumor size groups after PSM

After PSM					
Variables	C-index				
	ALL	T1N0M0	T2N0M0	T3N0M0	T4N0M0
Histology	0.508	0.514	0.504	0.507	0.52
Grade	0.511	0.516	0.507(p>0.05)	0.515(p>0.05)	0.547
Tumor size	0.519	0.543	0.517	0.512(p>0.05)	0.543(p>0.05)
T stage	0.533	-	-	-	-
Primary site	0.516	0.512	0.521	0.514	0.535
